# WeChat app combined CBL in oral medicine clinical training: A review

**DOI:** 10.1097/MD.0000000000033102

**Published:** 2023-03-17

**Authors:** Hong He, Jingyi Xu, Mingjie Sun, Jing Shao, Xiaotong Deng, Li Zeng

**Affiliations:** a The Affiliated Stomatology Hospital, Zhejiang University School of Medicine, Hangzhou, China; b Key Laboratory of Oral Biomedical Research of Zhejiang Province, Hangzhou, China.

**Keywords:** CBL, clerkship, clinical training, OPMDs, oral medicine, pre cancer, residents, WeChat app

## Abstract

Hotly used in student-centered medical education worldwide, case based learning (CBL) is worthen with WeChat, the most popular communication app and is widely used in all walks of life. We have practiced several years combining WeChat and CBL in the clinical training of oral medicine for young doctors, promoting outcomes over traditional bedside training. This article’s objective is demonstrating the acceptability and merits of WeChat CBL in the clinical training of oral medicine for young doctor. A total of eighty young doctors and 2 tutors participated in this study for interns of a every 2-month training during January 2018 to 2020. The control group used clinical bedside mode; the experimental group used bedside plus WeChat CBL mode. The evaluations included participation passion, daily routine and final test. Ten clerkships and thirty residents were in same number respectively of experiment and control groups. The participants in the experimental group produced a higher degree of participation in discussions. The twice and above Q&A action percentage is 40% in experimental group rather than 25% in control group. Daily assessment and final examination scores in the experimental group were significantly higher than those in the control group (*P* < .001). WeChat CBL mode has a positive effect on students’ learning enthusiasm, assessments and evaluations in clinical training of oral medicine.

## 1. Introduction

Oral medicine is a specialty of stomatology that is concerned with oral health problems including medical or psychological disorders and conditions affecting the oral and maxillofacial region. The instruction of oral medicine can be challenging for its comprehensive scope and heavy symptoms.^[1]^ While the boundary within the discipline seems obvious due to over subdivisions in specialized hospital, how to make systematic and comprehensive interrogation, reasonable consultation, make students grasp clinical medication effectively and master the oral status as causes of potentially malignant diseases are the key and difficult points in the clinical teaching of oral mucosa diseases. The practical training is a key component in oral medicine education, which is a teacher-centered process rather than a student-centered practice in tradition. And it leads to so major problems that students lack self-reliance, independence or diversity in solving clinical problems by themselves,^[2]^ with poor attendance and low levels of engagement in lecture-based instruction of over-presentation of knowledge.

Case-based learning (CBL) has been applied to medical education.^[3,4]^ This learning mode is applicable for the oral medicine practical training. And it differentiates from problem-based learning (PBL) with prior academic preparation, a more involved facilitator, and a focused learning trajectory.^[4,5]^ Meanwhile, WeChat app is a substantially popular and daily communicational contact application in China,^[6]^ which is convenient to upload pictures, speech or leave sonic words, share videos and documents, or hold a real-time video conferences. Almost all people are proficient in using it on Android, iPhone, BlackBerry, Windows and Symbian platforms. It has been used as the platform for online PBL in dental clerkships^[7]^ showing to us its feasibility in CBL teaching mode.

As an emerging educational model, CBL takes advantages of efficiency, balanced discussion and more feedback opportunities while there is not so much reports on its use in oral medicine, especially WeChat or WeChat CBL mode. The aim of this report was to observe the implementation and effectiveness of WeChat CBL mode in oral medicine practical training compared with usual way.

## 2. Methods

### 2.1. Participants

In this study, twenty participants were clerkship students and sixty were residents rotating at the department of oral medicine of the Affiliated Stomatology Hospital of Zhejiang University School of Medicine during Jan. 2018 and Jan. 2020, in China. During the clinical rotating, the students were randomly divided into 2 equal groups including students of clerkship as 10 and residents as 30 in same number respectively between experiment and control groups according to different clinical teaching mode. There is no statistically significant difference in the gender, age and educational level of 2 groups members. Kinds of real clinical cases were involved, including recurrent aphthous ulcer, oral lichen planus, oral leukoplakia, oral candidiasis, oral herpes simplex, bullous diseases and other common diseases of oral mucosa. Tutor in each group was assigned from clinical doctors with at least 1 year or more of experience in traditional CBL teaching.

Patient cases who have completed current treatment and did not relapse within 3 months were involved in the study. For each patient, their medical records are compiled into complete and individual medical reports. All participants and patients joined this study with informed consents, the latter have agreed their medical records to be shared as medical learning materials without their specific individual information such as names, address and etcetera. All tutors and students agree not to arbitrarily disseminate and disclose any form of case data.

### 2.2. Bedside learning mode

In this group, the learning mode was traditional and teacher-centered practical training. The students were taught by bedside instruction for case analysis learning. Teachers taught and answered questions to students regarding the types and conditions of patients with the oral mucosal diseases, including: general medical situation, disease history collection, physical examination, diagnosis, treatment and etcetera.

### 2.3. Bedside plus WeChat CBL mode

**Figure 1.**
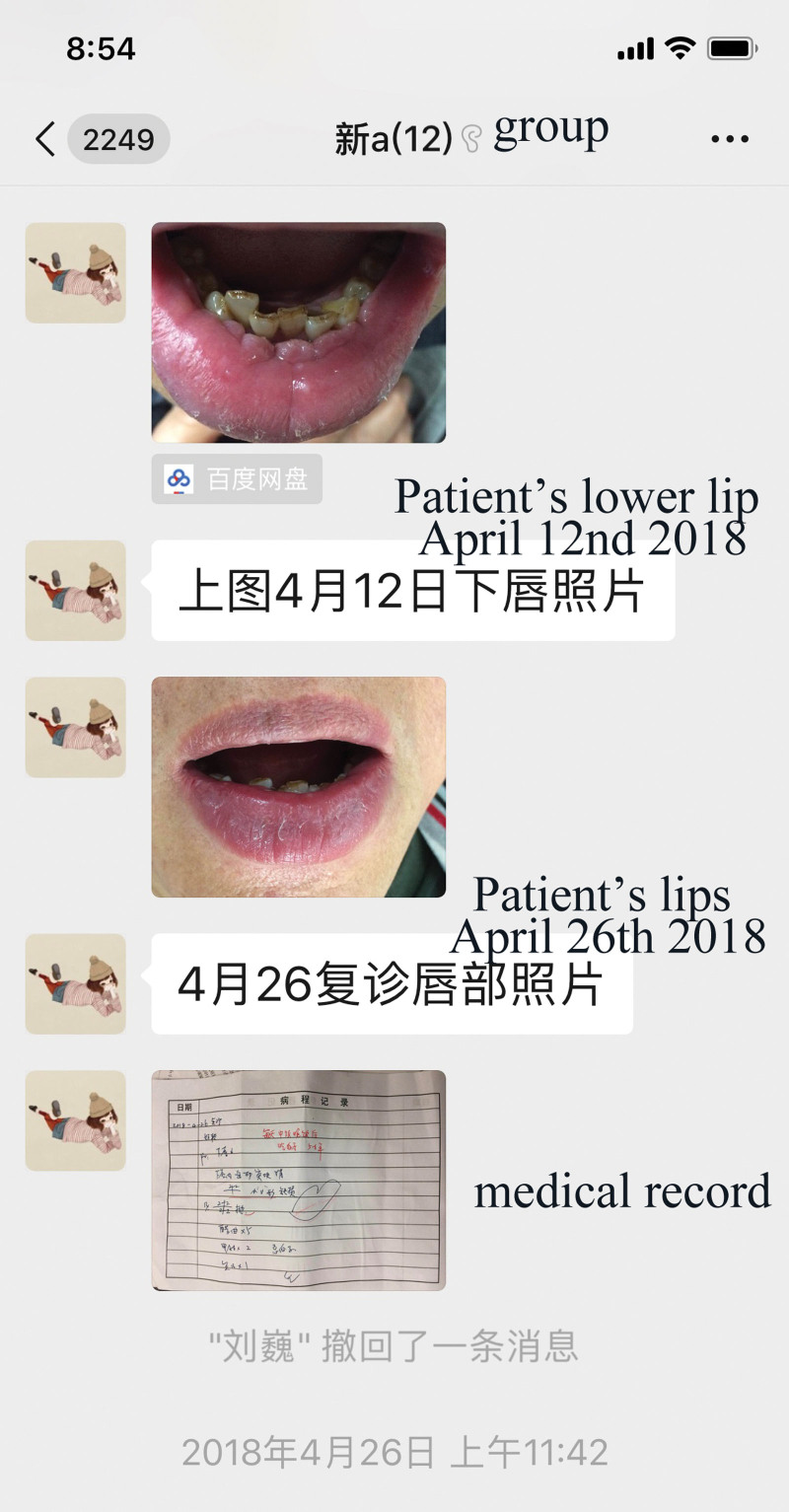
Tutor let assistant upload a CBL case to the WeChat CBL group. CBL = case-based learning.

**Figure 2.**
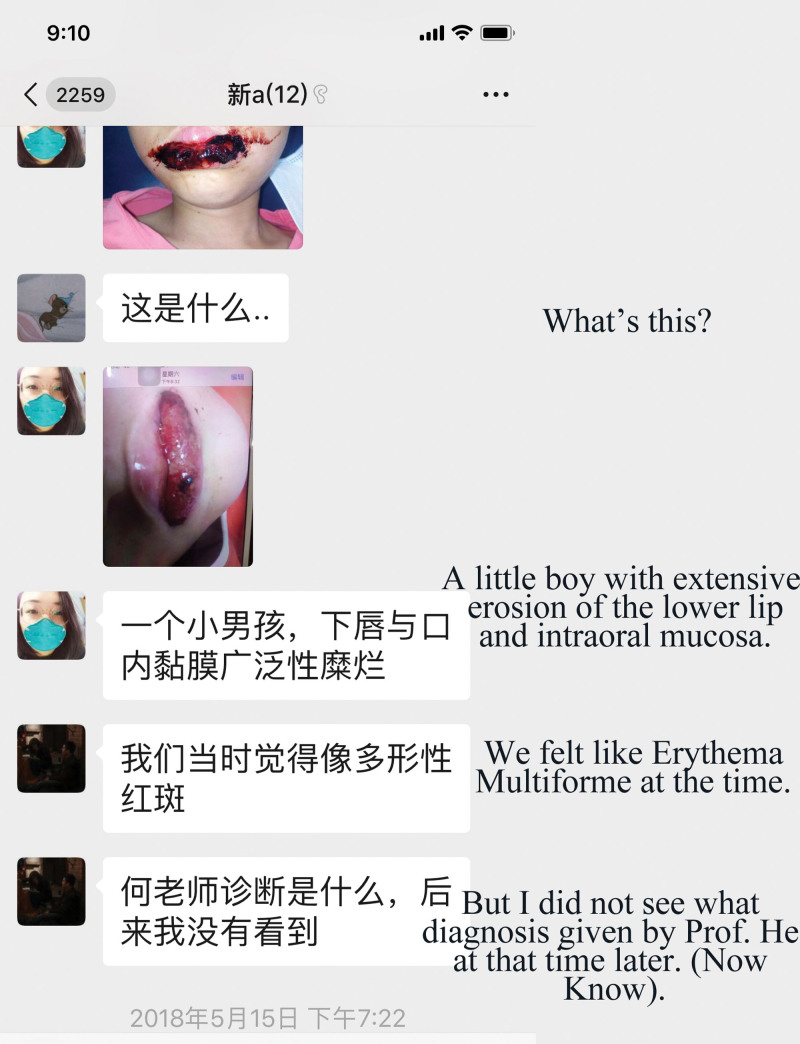
Students in the group have a discussion on the case.

**Figure 3.**
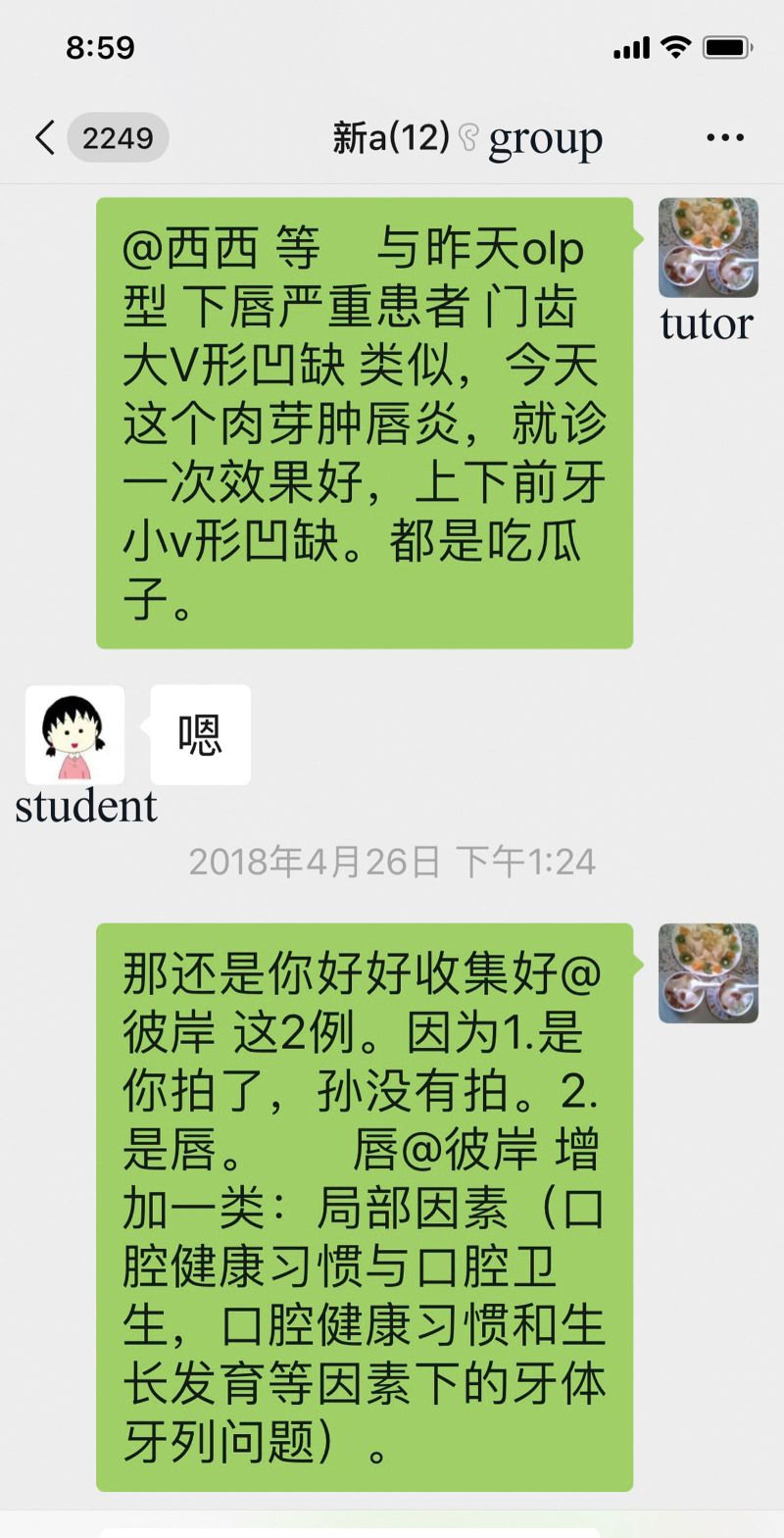
Tutors induced more and more knowledge with the discussion going on.

**Figure 4.**
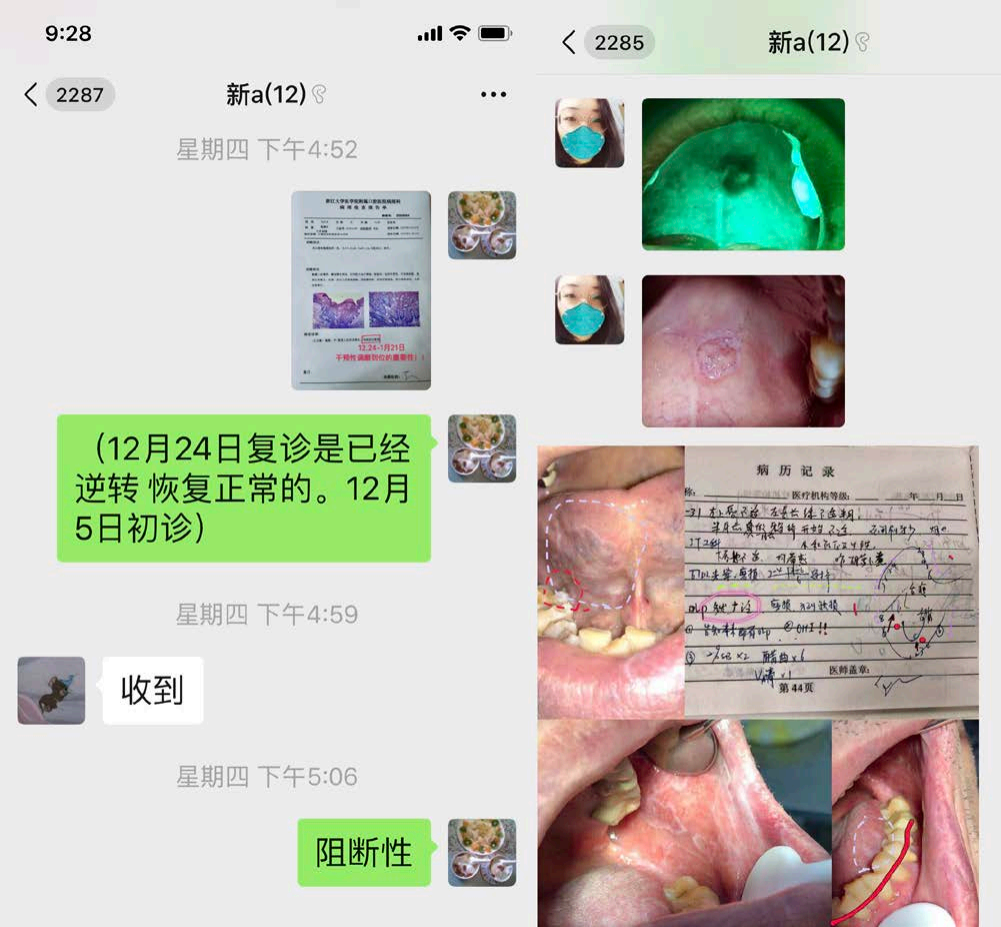
Students and tutors uploaded multifaceted and multidisciplinary documents to debate and study.

**Figure 5.**
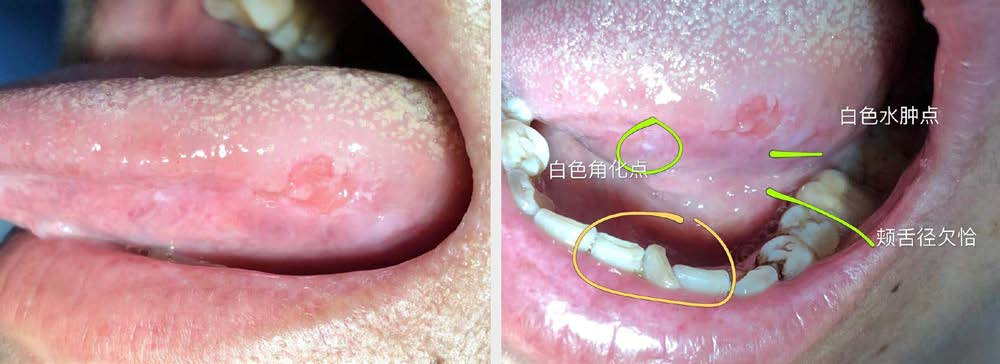
Tutors gave a picture on summary of the case at the end of learning.

WeChat application is widely used for daily contact in China and work communication. Some scholars have been using WeChat in medical teaching and doing well.^[8]^ So, it is very naturally for us to catch a glimpse on this basis using WeChat to provide the communication platform for CBL mode. The teachers in this group have been using WeChat in daily life for over 7 and 6 years for teaching. Besides routine bedside learning in clinic, this group of students accept WeChat CBL in addition. The tutor selected CBL cases and uploaded corresponding medical reports which they have prepared before to the WeChat CBL group (Fig. 1). The tutors and each student in the group could ask questions relative to the case (Fig. 2), and tutors would answer or induce more and more knowledge (Fig. 3). Students and tutors also could research and upload data associated with the case by image, text, voice, video, or other forms (i.e., documents) to debate or study (Fig. 4). Finally, tutors gave brief summaries of learning (Fig. 5).

### 2.4. Outcome evaluation

This study mainly evaluated the effects of the 2 learning modes by 2 aspects, whether students have effectively improved their learning motivation, knowledge mastery and clinical thinking ability, as well as their teamwork performance. The evaluations included 2 observations: learning motivation and participation passion: question raising times; knowledge mastery and clinical thinking ability: theoretical and practical skill examinations for oral medicine after the training. For every group, they will have 1 routine and 1 finial test. The routine test contained a medical record writing test and a clinical operation test. The final examination contained a theoretical written test and a case report. We developed such scoring standards to comprehensively evaluate students’ theoretical and clinical thinking skills.

### 2.5. Statistical analysis

Data were analyzed with SPSS 25.0 software (IBM Corp, Armonk, NY). Test scores and performance were collected and analyzed from 2 groups for further analysis. With the purpose of comparing the data between the 2 groups, Student *t* test was used. *P* value of < .05 was considered as significant.

## 3. Results

Eight students are involved in the study during January 2018 to 2020 and they are randomly and equally divided into 2 groups accepting bedside teaching or bedside teaching plus WeChat CBL. According to the statistics, the monthly Q&A frequency of the participants in the departmental vocational study was found that participants in the experimental group had a higher degree of participation in the discussion, and the detailed results are shown in Table 1.

**Table 1:** The monthly Q&A frequency of the participants in the departmental vocational study.

QA frequency (times)	The first month			The second month		
	Zero	Once	Twice and above	Zero	Once	Twice and above
Control group (person, %)	19/40 (47.5%)	10/40 (25%)	11/40 (27.5%)	11/40 (27.5%)	19/40 (47.5%)	10/40 (25%)
Experimental group (person, %)	9/40 (22.5%)	21/40 (52.5%)	10/40 (25%)	4/40 (10%)	20/40 (50%)	16/40 (40%)

By comparing the examination performances of the 2 groups’ participants: the daily assessment and final examination scores of the participants in the experimental group were found significantly better than those in the control group (*P* < .05). Detailed results are shown in Tables 2 and 3.

**Table 2:** Routine assessment results (*X̅* ± *S*, score).

n = 40 respectively	Medical record writing (totally 50 scores)	Clinical operation (totally 50 scores)
Control group	32.80 ± 2.89	35.08 ± 3.15
Experimental group	46.10 ± 4.86	44.45 ± 2.92
*T* value	14.876	13.797
*P* value	<.001	<.001

**Table 3:** Final examination results (*X̅* ± *S*, score).

n = 40 respectively	Theoretical test score (totally 50 scores)	Case report (totally 50 scores)
Control group	36.05 ± 2.693	36.10 ± 2.71
Experimental group	45.7 ± 2.37	45.32 ± 1.632
*T* value	17.013	18.433
*P* value	<.001	<.001

## 4. Discussion

The results show that the participants in the experimental group produced a higher degree of participation in discussions. The percentage of twice and above Q&A frequencies is 40% in experimental group rather than 25% in control group. Daily assessment and final examination scores of the participants in the experimental group were significantly better than those in the control group (*P* < .001). Based on this, we briefly analyzed the current clinical teaching situation of oral medicine in China and the benefits that WeChat CBL can bring to us, as well as limitations.

### 4.1. Existence of dilemma in clinical teaching

#### 1.4.1. Dilemma in oral medicine teaching.

Oral mucosal diseases are characterized by complex etiology, multiple clinical lesions and close involvements with systemic diseases.^[1,9]^ The clinical diagnosis and treatment of difficult cases need the joint consultation of pathology, oral surgery, skin and endocrine. Furthermore, the time duration of standardized training for oral medicine department is fixed and short, usually only 1 to 2 months, which is a challenge for teachers to achieve the teaching goal and improve students’ clinical ability like diagnosis level. Weak cross-cooperation in specialized hospital also lead to poor overall view of the diseases.

Due to its complexity, students usually fail to recognize and differentiate lesions when they have few experience in clinic. It is essential to spend a tremendous amount of time in learning in clinic while the rotation time of oral medicine department is fixed and short. In this context, we hope to find a platform that can help students see more clinical cases in the fixed rotation time and communicate with teachers in a timely manner.

#### 2.4.1. New challenge for clinical teaching mode.

In clinical training, most of the students are undergraduate and graduate students just out of the school. The students’ personality and self-awareness are stronger, and their learning ability may vary greatly. The development of clerkship in different colleges and universities is different, and the degree of students’ mastery of clinical skills is uneven. With the development of IT and diversification of learning methods and contents, the access to knowledge is no longer limited to a single textbook, and students lack the ability to identify the useful knowledge for themselves quickly and efficiently. Undertaking different degrees of clinical, scientific research and teaching tasks, although the residents teaching team is composed of senior doctors with more than 5 years of clinical experience as attending physician, deputy directors and directors, it is still difficult to “teach students in accordance with their aptitude” in a limited period of time, and make timely and effective responses to the students’ common and individual problems, so as to realize the interacting mode of “both teachers and friends” between teachers and students.

Stomatology residents teaching is mainly in out-patient clinic, the number of outpatients is large in China, the clinical time for per patient is compressed. Due to imbalance ratio between patients and doctors, students and teachers faced with heavy clinic work. Although helping students quickly familiarize themselves with various practice, there is the problem of reduction of thinking and discussion time, which is easy to cause thinking limitations and reduce the benefit of ideal treatment and teaching.^[10]^ Heavy clinical work and boring training mode can easily lead to negative emotions such as burnout of students and reduce their enthusiasm for learning.^[11]^ And patients acquiring so-called “professional knowledge” and “scientific opinions” through a variety of network channels, plus a strong legal sense of self-personality, are more riskily and possibly hostile and uncooperative to junior doctors, so students are unable to obtain relatively systematic and comprehensive information for diagnosis and treatment, thus affecting the fulfillment of addressing plan and the development of clinical career. Teachers need to spend time explaining to patients, which reduces the time of teaching. Medical students face the same confusion when they go into clinical work in the future. It is worth pondering for residents and teachers about how to deal with the challenges such as the highly sensitive state of some patients, diverse clinical skills with technologies and emotional quotient, so as to achieve harmonious development between doctors and patients, teachers and students.

According to past studies, WeChat has been used in mobile teaching like WeChat PBL teaching^[12]^ and be acknowledged as a free, interactive, adaptable, sustainable and more participatory teaching style.^[13]^ Thus, WeChat CBL is nicely facilitated with these reality situations.

### 4.2. Advantages of WeChat CBL

#### 1.4.2. Relatively diverse content and forms.

The traditional bedside teaching provides a true feeling and real situation, which can obtain the first-hand clinical data recording the disease text and the patient’s physical signs, but the teaching content is relatively limited under clinical situation. WeChat platform has been used to carry out a variety of teaching forms, including text, audio, video, charts, animation and so on, receiving a high degree of acceptance, and presented obvious advantages in promotion of theoretical knowledge evaluation (*P* < .001) and case report assessment in session examination (*P* < .001). Students take what they have seen and heard as a starting point, under the guidance of teachers, collect scattered and diverse disease data, into short & simple theory and case review easy to understand^[14,15]^ and share with others by discussion and practice. Further adjustment and supplement according to feedback to consolidate learning achievements, delivered a higher degree of standardization in the writing of daily medical records and clinical practice (*P* < .001). WeChat platform combined the bedside teaching realize the virtuous circle of theory and practice, inheritance and innovation, clinical and scientific research, and transform the stage modular learning into a continuous and systemically style.

#### 2.4.2. Relatively flexible time.

**Figure 6.**
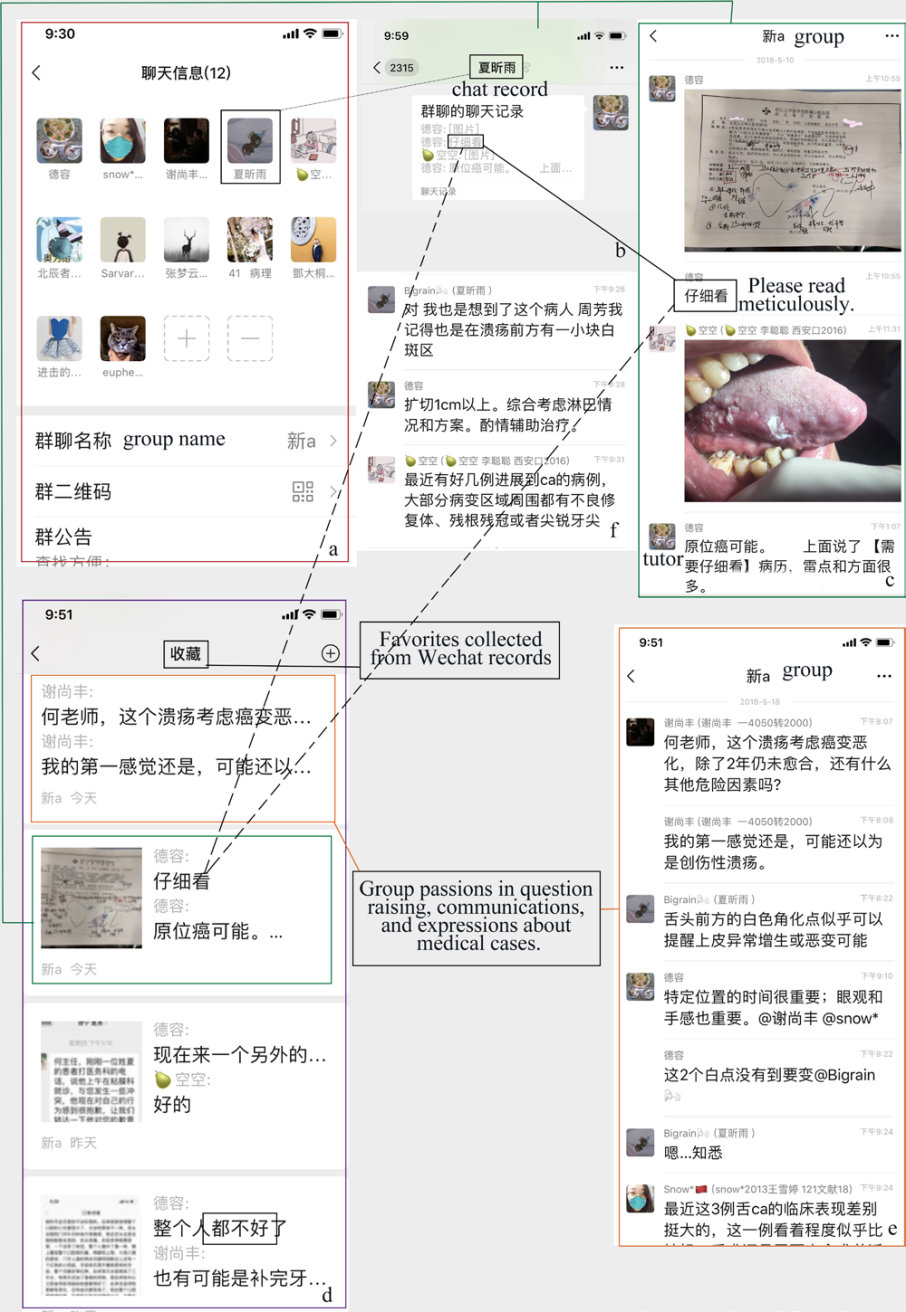
(A–C) Favorite content in collection can be shared or resent to single or group members to review in style of chat record. (D–F) Topic-oriented collection of WeChat record is convenient for afterwards study and review.

The traditional bedside teaching is mostly concentrated in some specific time of outpatient clinic, and the discussion of difficult cases is limited to the clinic time. In special condition, it is necessary to take into account both patients and family members, the depth and breadth of discussion is limited, and the participation of many students with weak foundation is not high. Most make teaching to be the top or un-top student’s passive participation. With the help of WeChat platform, clinical teaching with interactive learning can be extended to any time or place outside the outpatient clinic, the timeliness of releasing new trends, important notices and other information is more than 24 hours or even longer (Fig. 6). The easy spending of leisure time can play a role in urging the culturing of learning habits and in realizing the smooth transition from the school to the clinic.

#### 3.4.2. Relatively comfortable relationship.

The multi-way interaction in WeChat group improves smoothness of the relationship between teachers and students, students and students, doctors and patients. Extensive communication content can achieve all-round teaching of clinical skills and humanistic literacy for students. Through this communication it is easy to create a relaxed working and learning atmosphere, understanding students’ personality, clinical ability, psychological dynamics, reducing patient’s possible estrangement and distance, reducing burnout, and improving their learning enthusiasm.^[16]^ In this study, the experimental group’s participation in topic discussion of the monthly Q&A in the departmental vocational study was significantly higher than that in the control group (Table 1). The use of stage-by-stage teaching can help students who with weak foundation and low learning ability to adapt to the teaching progress, complement each other’s advantages and unite with each other. For students who finish the course and move into his career work after standardized training, this new mode extends the teaching time to the exchange of cross-disciplines and realize more continuing education indirectly.

Overall, clinical teachers integrate and identify a large number of resources to screen out useful, real, reliable and advanced professional knowledge to the students. While answering the questions of students and patients, they can improve themselves, find innovation, promote the development of subject diagnosis and treatment technology and get new research ideas. Tracing the outcome of patients’ condition through the platforms is helpful to understand the general and individual characteristics of a disease, with time for space convenience, also with space for time convenience, and optimize the patient administration, teaching administration and disease administration, and improve for any new problems. Prospectively under the background of modern information resources, it allows us to highlight new emphasis on focusing back to emotional intelligence and humanity development,^[17]^ which can be more important in some aspects than just focusing on technology ability, and is being hopeful to be reported in another article.

### 4.3. Limitations

Although the application WeChat has been used for daily communication widely in China, combining it with dental clerkship and CBL teaching has less amount of probands. We use question raising times as evaluation index of learning motivation and participation passion which is convenient to conduct statistics but it may be influenced by some factors such as different personalities of students. Using WeChat as the teaching platform may also cause some unconscious students to more conveniently not participate in the discussion than face-to-face teaching.

## 5. Conclusions

WeChat CBL assisted bedside training not only takes the advantage of convenience and sharing multifaceted and multidisciplinary materials, but also makes students easy to do retrospective learning out of the clinic.

It broadens students’ learning enthusiasm and improves level of knowledge and clinical capability. Besides flexibility for study, training, teaching and patients’ privacies, it offers comfortable relationships and administrations among students, teachers, leaders, doctors and patients. Prospectively, emotional quotient and humanity development in medical practice are also our training items besides professional knowledge by WeChat CBL to be reported and shared.

## Author contributions

**Conceptualization:** Hong He.

**Data curation:** Hong He.

**Draft manuscripting supervision and revisions:** Hong He.

**Writing – original draft:** Hong He, Jingyi Xu, Mingjie Sun, Jing Shao, Xiaotong Deng, Li Zeng.

**Writing – review & editing:** Hong He, Jingyi Xu, Mingjie Sun, Jing Shao, Xiaotong Deng, Li Zeng.
